# Important Mycoses of Wildlife: Emphasis on Etiology, Epidemiology, Diagnosis, and Pathology—A Review: PART 1

**DOI:** 10.3390/ani12151874

**Published:** 2022-07-22

**Authors:** Iniobong Chukwuebuka Ikenna Ugochukwu, Chioma Inyang Aneke, Nuhu Abdulazeez Sani, Jacinta Ngozi Omeke, Madubuike Umunna Anyanwu, Amienwanlen Eugene Odigie, Remigius Ibe Onoja, Ohiemi Benjamin Ocheja, Miracle Oluchukwu Ugochukwu, Iasmina Luca, Olabisi Aminah Makanju

**Affiliations:** 1 Department of Veterinary Pathology and Microbiology, University of Nigeria, Nsukka 410001, Nigeria; iniobong.ugochukwu@unn.edu.ng (I.C.I.U.); chioma.aneke@unn.edu.ng (C.I.A.); jacinta.omeke@unn.edu.ng (J.N.O.); madubuike.anyanwu@unn.edu.ng (M.U.A.); remigius.onoja@unn.edu.ng (R.I.O.); 2 Department of Veterinary Medicine, University of Bari, 70010 Bari, Italy; amienwanlen.odigie@uniba.it; 3 Department of Veterinary Pathology, University of Abuja, Abuja 900109, Nigeria; nuhu.sani@uniabuja.edu.ng; 4 Department of Veterinary Public Health and Preventive Medicine, University of Benin, Benin City 300238, Nigeria; 5 Department of Biosciences, Biotechnologies and Biopharmaceuticals, University of Bari, 70010 Bari, Italy; ohiemi.ocheja@uniben.edu; 6 Department of Veterinary Physiology and Biochemistry, University of Benin, Benin City 300238, Nigeria; 7 University of Nigeria Medical Centre, Nsukka 410001, Nigeria; miramimiugochux@gmail.com; 8 Department of Pathological Anatomy and Forensic Medicine, Faculty of Veterinary Medicine, Banat’s University of Agricultural Sciences and Veterinary Medicine, 300645 Timișoara, Romania; 9 National Veterinary Research Institute, Vom 930010, Nigeria; olabisimakanju@yahoo.com

**Keywords:** wildlife, fungal diseases, zoonosis, diagnosis, pathology

## Abstract

**Simple Summary:**

The number of wild animals is steadily declining globally, so the early diagnosis and proper treatment of emerging diseases are vital. Fungal diseases are commonly encountered in practice and have a high zoonotic potential. This article describes aspergillosis, candidiasis, histoplasmosis, cryptococcosis, and penicilliosis, and is only the first part of a detailed review. The laboratory methods (fungal isolation, gross pathology, histopathology, histochemistry, cytology, immunohistochemistry, radiography, CT, PCR, or ELISA) used in the diagnosis and the clinical details that provide a complete view of the mycoses are presented.

**Abstract:**

In the past few years, there has been a spurred tripling in the figures of fungal diseases leading to one of the most alarming rates of extinction ever reported in wild species. Some of these fungal diseases are capable of virulent infections and are now considered emerging diseases due to the extremely high number of cases diagnosed with fungal infections in the last few decades. Most of these mycotic diseases in wildlife are zoonotic, and with the emergence and re-emergence of viral and bacterial zoonotic diseases originating from wildlife, which are causing devastating effects on the human population, it is important to pay attention to these wildlife-borne mycotic diseases with zoonotic capabilities. Several diagnostic techniques such as fungal isolation, gross pathology, histopathology, histochemistry, cytology, immunohistochemistry, radiography, CT, and molecular methods such as PCR or ELISA have been invaluable in the diagnosis of wildlife mycoses. The most important data used in the diagnosis of these wildlife mycoses with a zoonotic potential have been re-emphasized. This will have implications for forestalling future epidemics of these potential zoonotic mycotic diseases originating from wildlife. In conclusion, this review will highlight the etiology, epidemiology, diagnosis, pathogenesis, pathogenicity, pathology, and hematological/serum biochemical findings of five important mycoses found in wild animals.

## 1. Introduction

Although human and non-human hosts are increasingly becoming prone to infectious spores, fungi have been omitted as a possible cause of diseases [1,2]. However, a growing number of the stubborn fungal diseases of animals caused by cosmopolitan and pathogenic fungi have occurred over the last two decenniums [3]. This has caused an atypical total of fungal and fungal-like diseases that have recently caused some of the utmost austere wild animal extinctions ever reported [2,3,4].

Some zoonotic mycoses of wild animals can cause significant public health problems. Additionally, the appreciable tally of zoonotic mycoses is among the most representative and frequently diagnosed fungal diseases globally [5]. From a universal perspective, zoonotic infections have been identified for many centuries, and they account for the majority of emerging and re-emerging infectious diseases that have significantly impacted the health status and economy of countries around the globe [6]. Viral and bacterial zoonotic diseases originating from wildlife have continued to emerge and spread rapidly, with some of them surpassing epidemic levels to pandemic proportions [7,8,9]. Little attention has been paid to fungal zoonoses originating from wildlife because of the low spread. In addition, an increase in their prevalence has been reported in the human population probably due to antibiotic resistance. As these organisms evolve, they may acquire the potential to cause epidemics; thus, there is a need to highlight these mycoses in wildlife.

The early diagnosis of mycoses is very important to be able to institute effective therapy. Universal methods for the laboratory diagnosis of mycotic infections include direct microscopic examination, histopathology, microbial culture, antigen detection, serology, and molecular tests [10]. Advantageously, a histopathologic examination detects fungal agents in invaded tissues and vessels. Additionally, it is also useful for detecting the host’s reaction to the fungus [11]. Thus, this technique, in addition to alternative methods (such as immunohistochemistry, in situ hybridization, and PCR), has been used to determine specific fungal agents present in histopathologic specimens [11,12,13]. Furthermore, techniques such as magnetic resonance imaging (MRI) and laser microdissection have provided useful results even in cases of mixed fungal infections [11,14].

Amongst the mycotic diseases of wildlife, aspergillosis is the major type of mycosis affecting wild animals, especially birds [15]. Candidiasis is a sporadic mycosis of wild birds and other animal species, but it differs greatly from aspergillosis by being transmitted by ingestion instead of inhalation [16]. In this review, in addition to aspergillosis and candidiasis, we will also discuss the etiology, transmission, epidemiology, diagnosis, pathology, and impact of some other important mycoses in wildlife such as histoplasmosis, cryptococcosis, and penicilliosis. Understanding the etiology, transmission, epidemiology, diagnosis, pathology, and impact of fungal diseases in wildlife is important for conceiving preventive strategies, treating associated diseases, and reducing public health risks.

## 2. Aspergillosis

Aspergillosis, also called brooder pneumonia, pseudotuberculosis, “asper” mycosis, or mycotic pneumonia is an important mycosis in wildlife chronicled many decades ago, but it remains a leading etiology of mortality in wild animals. Aspergillosis is predominantly a disease of the respiratory tract in animals, and it often becomes generalized leading to a variety of manifestations ranging from acute to chronic infections [16,17]. Although tissue predilection is flexible among species [16], it is broadly accepted that defective immunity and the inhalation of a sizable number of spores are important causative factors [16,17]. Aspergillosis is not a contagious disease, but it runs an acute course that rapidly results in fatality or persists as a chronic disease. Notably, some *Aspergillus* species are toxicogenic-producing aflatoxins [16].

### 2.1. Etiology

Aspergillosis is caused by saprophytic fungi species in the genus *Aspergillus*. *Aspergillus fumigatus* is recognized as the most important causative agent of aspergillosis [17]. *Aspergillus flavus*, *Aspergillus niger*, *Aspergillus glaucus*, *Aspergillus nidulans*, and other *Aspergillus* species have been isolated from single cases and/or mixed infections [17,18,19,20].

### 2.2. Epidemiology

All birds are susceptible to and a wide range of wild birds have died from aspergillosis. The earliest description of aspergillosis in wild birds was in a scaup (*Aythya marila*) in 1813 and a European jay (*Garrulus glandarius*) in 1815. It was also commonly associated with the deaths of loons (*Gavia* sp.) and marine birds, captive raptors, and penguins in zoological parks and other facilities [15,16]. Young birds appear to be more susceptible to aspergillosis than their adult counterparts. The distribution of aspergillosis in wild birds has a global reportage. The predisposing factors to aspergillosis include a high concentration of *Aspergillus* spores in the environment, humidity, poor ventilation [21,22], poor sanitation [23], and the prolonged storage of feed [17,24], which may increase the number of spores in the air. Factors including impaired immunity, an inadequate diet, metabolic bone diseases, toxicosis, shipping, quarantine or captivity, overcrowding, starvation, thermal discomfort, and animal migration can also result in a predisposition to mycosis [17,25,26,27,28,29,30]. *Aspergillus fumigatus* has been identified in wild boars in Bulgaria [31] and other *Aspergillus* species have also been reported in female killer whales (*Orcinus orca*) [32].

### 2.3. Diagnosis

The diagnosis of aspergillosis is problematic because its clinical signs are non-specific. Moreover, no single test provides a definitive diagnosis. Therefore, its diagnosis is dependent on an accretion of empirical evidence from the history, clinical signs, hematology, biochemistry, serology, radiographic changes, endoscopy, and culture of the fungus [17,33,34].

The diagnosis pf aspergillosis is centered on detecting pathognomonic lesions and isolating the fungus from the tissues. A definitive diagnosis requires a demonstration of the organism in the tissue by cytology or histopathology and a histochemical identification using special stains such as periodic acid-Schiff (PAS) [16]. The hostile *Aspergillus* species cannot be identified by only the isolation and description of its colonial morphology through an agar culture [16,35]. Histopathological lesions can be suggestive but not pathognomonic due to the high similarity of the in vivo hyphae and their in situ manifestations [17]. Hence, histopathology is not the best technique for identifying fungal species [36,37]. Thus, a confirmatory diagnosis of the etiology could be achieved by immunohistochemistry [17,38,39]. Serum protein electrophoresis is also a diagnostic tool for diagnosing aspergillosis in birds. Nevertheless, different polymerase chain reaction (PCR) assays (including real-time polymerase chain reactions) have been used for diagnosing avian aspergillosis in body fluids, respiratory tract granulomas, and tissue (biopsy) samples [37].

Serological tests have been developed to confirm an early and more definite diagnosis of aspergillosis [40], but in cases of immunosuppression, with the resultant low antibody production, there are false-negative results. However, the detection of antibodies can be useful in chronic cases since antigen levels may be low [33]. Several serological test methods such as counter-immunoelectrophoresis, agar gel immunodiffusion, and enzyme-linked immunosorbent assays have also been proven useful, although the positive tests are only counted valid when supported by other evidence [37,40,41,42]. Imaging techniques such as radiography and computed tomography can also be used [43]. Nonetheless, even with these imaging tests, the diagnosis of aspergillosis still requires identification by biopsy, smear, or culture [21].

### 2.4. Pathology

#### 2.4.1. Pathogenicity and Pathogenesis

*Aspergillus* species secrete copious secondary metabolites called mycotoxins [43,44]. Mycotoxins, produced during enzymatic reactions, are one of the reputed virulence factors of *Aspergillus* utilized in subduing the host’s immunity, thereby encouraging the infectivity of the fungus [43,45]. *Aspergillus* produces some outstanding mycotoxins including aflatoxins, gliotoxin, ochratoxin A, helvolic acid, ribotoxins, and so on [43,46,47].

The acute form of aspergillosis has caused devastating losses. Inhaled *Aspergillus* spores activate a cellular response in the lungs followed by airway blockage with cellular debris and fungal filaments. Asphyxiation quickly follows and ultimately leads to death [16].

However, aspergillosis may also result from an allergic reaction to the inhaled conidia [22,48]. This gradually but unremittingly diminishes respiratory function since the lungs and air sacs are infected in the chronic forms of aspergillosis. Consequently, there is a subsequent mycotic dissemination to the liver, gastrointestinal tract, and viscera [16].

#### 2.4.2. Clinical Signs

Aspergillosis’ clinical manifestations are hinged on the infective dose, spore distribution, pre-existing diseases, and the immune response of the host [49]. Although aspergillosis is predominantly a disease of the respiratory tract, any other body system can be affected [50]. The typical signs in wild birds include emaciation, dyspnea (primarily characterized by gaping or rapid opening and closing of the bill), unthriftiness, drooping wings, exercise intolerance, depression, weakness, and anorexia [19,51]. Perelman and Kuttin (1992) observed depression, lethargy anorexia, stagnant growth, and high mortality in ostriches affected by aspergillosis [19].

An aspergillosis infection in the brain can result in a loss of muscular coordination and torticollis [19]. Other neurological signs associated with aspergillosis include ataxia, seizures, or a loss of equilibrium, and all of these indicate the central nervous system’s (CNS) involvement. Additionally, signs such as head shaking and tail “bobs” have also been reported [23,35,52]. Furthermore, a granulomatous extension from the caudal air sacs to the spine or sacral plexus can cause partial or total paresis or paralysis [23,26,52,53].

#### 2.4.3. Macroscopic Findings

Exudative rhinitis has been noted in wild birds suffering nasal aspergillosis [50]. Birds with chronic aspergillosis present lesional *foci* of different sizes in their lungs and air sacs. Typically, these lesions are flattened yellow plaques with a cheesy appearance and consistency [16]. The lesions and extensive fungal growth may fully border the air sac presenting a “bread mold” appearance. In acute aspergillosis, the birds are usually in a good body condition; however, the air sacs are thickened, with the most striking type of lesion presenting a lung that is firm and dark red with multifocal small yellow nodules [16]. Perelman and Kuttin (1992) observed that ostriches (*Struthio camelus*) with a concurrent infection of *A. niger* and *A. flavus* were severely emaciated with softened bones and distorted thoracic cages [35]. There were diffused cream-colored nodules throughout the lungs, and in some of the birds the ribs were locally thickened with nodulation on the pleura. Furthermore, some of the birds also presented thickened air sacs with yellowish patches. Granulomatous nodules were also observed mainly on the air sacs and lungs of Magellanic penguins (*Spheniscus magellanicus*) [54]. In wild Eurasian black vultures (*Aegypius monachus*), severe granulomatous pneumonia and serofibrinous pleuropneumonia were observed [55].

In female killer whales (*Orcinus orca*), a diffused fungal pyogranulomatosis was distinguished with a distribution in the lymph nodes (mandibular and visceral), heart, and lungs [32].

*Aspergillus* blepharitis and dermatitis involving the eyelids and the head have been described in a peregrine falcon–gyrfalcon hybrid (*Falco peregrinus*–*Falco rusticolus*) [29]. In this case, there was a right ventricular dilatation due to pulmonary hypertension, with or without ascites, and pneumocongestions caused by ventricular failure were identified in wild birds [17]. Cheesy plaques were seen around the nictitating membrane [16].

#### 2.4.4. Histopathological Findings

Histological lesions in wild Eurasian black vultures (*Aegypius monachus*) included pyogranulomatous pneumonia and suppurative parabronchiolitis/pleuritis/air sacculitis with some septated fungal hyphae [55]. Whereas a histology of the lungs in ostriches (*Struthio camelus*) and Magellanic penguins (*Spheniscus magellanicus*) showed a subacute to chronic granulomatous pneumonia with many multinucleated giant cells, necrotic areas, and many PAS-positive hyphae with diploid branching [19,54]. Thrombosis and a fungal hyphae invasion in blood vessels, bronchitis with a hyphal invasion of the bronchial wall, and the presence of a few *Aspergillus* fruiting heads were present [19]. Meningoencephalitis with multiple foci of necrosis has been reported for eider ducklings (*Somateria mollissima*) as a result of aspergillosis [19]. A histology of the ribs showed osteomyelitis with osteonecrosis, the presence of infiltrating inflammatory cells, and septated hyphae [19]. Upon a special histochemical staining, nodular reactions along with fungal spores and a characteristic radiating club, diploid branching septated hyphae, and mycelial conidiophores were observed in wild species including Rhesus monkeys (*Macaca mulatta*), sambar deer (*Rusa unicolor*), striped hyena (*Hyaena hyaena*), gayal (*Bos frontalis*), East African oryx (*Oryx beisa*), waterbuck (*Kobus ellipsiprymnus*), and greater kudu (*Tragelaphus strepsiceros*) suffering aspergillosis [34].

#### 2.4.5. Hematological Findings

A hematological analysis in a gyrfalcon (*Falco rusticolus*) showed leukocytosis, heterophilia, monocytosis, and thrombocytosis [51].

## 3. Candidiasis

Also known as moniliasis, thrush, or sour crop, candidiasis is an infrequent opportunistic fungal disease of importance in parakeets (*Melopsittacus undulatus*). It is a gastrointestinal infection in numerous species of wild birds raised in captivity [56]. There are also reports of candidiasis in different wild animals such as bears (*Ursus* sp.), European beavers (*Castor fiber*), dolphins (*Delphinus delphis*), baboons (*Papio* sp.), cheetahs (*Acinonyx jubatus*), tortoises (*Chelonoidis* sp.), guanacos (*Lama guanicoe*), chimpanzees (*Pan troglodytes*), monkeys, gorillas (*Gorilla gorilla*), wild porcupines (*Erethizon dorsatum*), and so on [57,58,59,60,61,62,63,64,65,66,67].

### 3.1. Etiology

*Candida albicans* is the substantial *Candida* species causing candidiasis or candidosis [56,68]. *C. albicans* is a normal inhabitant of the human alimentary canal, as well as that of many species of lower animals [16,56,68]. Other *Candida* species such as *C. glabrata*, *C. krusei*, *C. parapsilosis*, *C. metapsilosis*, *C. tropicalis*, *C. guilliermondii*, and *C. auris* have also been isolated [69,70].

### 3.2. Epidemiology

Wild animal species reported to have been affected by *Candida* infection include bears (*Ursus* sp.), beavers (*Castor fiber*), wild boars (*Sus scrofa*), dolphins (*Delphinus delphis*), baboons (*Papio* sp.), cheetahs (*Acinonyx jubatus*), tortoises (*Chelonoidis* sp.), guanacos (*Lama guanicoe*), chimpanzees (*Pan troglodytes*), monkeys, gorillas (*Gorilla gorilla*), wild porcupines (*Erethizon dorsatum*), and free-ranging wild birds such as canaries (*Serinus canaria*), macaws (*Ara macao*), and cockatiels (*Nymphicus hollandicus*) [16,70]. Candidiasis is an occasional disease of importance that is considered a disease or an intestinal infection in numerous species of wild birds being raised in captivity [16,69,71]. Candidiasis has a worldwide reportage but there is no known seasonal incidence [16,56]. However, young animals, especially birds, are generally more susceptible to infection. The ingestion of *C. albicans* in food or water is the conventional means for its transmission [16,56].

*C. albicans* is a common environmental organism and an opportunistic pathogen with the avian crop as its natural habitat [68]. Candidiasis has been observed in pigeons (*Columba livia domestica*), geese (*Anser*/*Branta* sp.), guinea fowl (*Numida meleagris*), pheasants (*Phasianus colchicus*), quails (*Coturnix coturnix*), parrots (*Psittacus* sp.), and other birds [68]. Contaminated litter and areas contaminated with human waste are potential sources of the acquisition of *Candida* [16,56]. *Candida glabrata* was isolated from thrush-like lesions in yellow-naped Amazon parrots (*Amazona* leucocephala), ring-necked doves, macaws, and cockatiels, while *C. krusei* was recovered from an Eclectus parrot (*Eclectus roratus*) suffering acute necrotizing ventriculitis [69].

### 3.3. Diagnosis

*Candida* spp. can be diagnosed by pathological (clinical, hematological, gross, and histopathological), radiographical, cytological, and microbiological evaluations [11,64]. PCR, ELISA, diagnostic biomarkers, and pan-fungal β-d-glucan have been used in the diagnosis of candidiasis, but these techniques appear to lack standardization, sensitivity, or specificity. Blood cultures remain the gold standard for the diagnosis of candidiasis; however, the test sensitivity of blood cultures is impecunious and dawdling [47,72].

### 3.4. Pathology

#### 3.4.1. Pathogenicity and Pathogenesis

The pathogenicity of *Candida* species is credited to certain virulence factors that assist pathogenic capabilities such as host recognition (which enables the pathogen to bind to host cells), host defense-evasion capabilities, adherence, biofilm formation (on host tissue and abiotic surfaces), the production of tissue-damaging degradative and hydrolytic enzymes (such as proteases, phospholipases, and hemolysin), yeast-to-hypha transition, contact sensing, thigmotropism, phenotypic switching and a range of fitness attributes [73,74]. The majority of *C. albicans* infections are associated with a biofilm formation on the host or abiotic surfaces. A biofilm confers tolerance to antimicrobials, which jeopardizes therapy even with recent therapeutic antifungal agents [27]. Thus, the pathogenicity of biofilm-producing *C. albicans* strains may be exacerbated, potentially resulting in high morbidity and mortality.

#### 3.4.2. Clinical Signs

There are no unique signs of *Candida* infection in field practice. However, various clinical signs, including stunted growth, listlessness, ruffled feathers, inappetence, dullness, passing greenish diarrhea, whitish plaques in the mouth, regurgitation, and weight loss, have been observed in affected wild birds [16,56]. A male myna bird (*Acridotheres tristis*) with systemic candidiasis and fungal osteoarthritis was depressed, lethargic, cachexic, and had inflamed foot joints [75]. Non-albicans *Candida* species were isolated from six wild birds that were diarrheic and suffered from regurgitation and melena [69]. An adult female Greek tortoise (*Testudo graeca*) with severe unilateral pulmonary candidiasis showed signs of dyspnea, lethargy, and anorexia [64]. A male Rhesus monkey (*Macaca mulatta*) with thrush showed lethargy, anorexia, and diarrhea [36]. Schmidt and Butler (1970) noted anorexia and diarrhea accompanied by severe dehydration in a baby chimpanzee (*Pan troglodytes*) [58]. La Perle et al. (1998) reported the involvement of the spleen, liver, kidneys, and lymph nodes in a geriatric captive cheetah (*Acinonyx jubatus*) diagnosed with systemic candidiasis [63]. The cheetah had a protracted *Helicobacter acinonyx*-associated intermittent chronic gastritis and chronic renal failure. These comorbidities possibly suppressed the animal’s immune system, thereby allowing *Candida* to flourish.

#### 3.4.3. Macroscopic Findings

The lesions of candidiasis in wild birds are mainly restricted to the upper areas of the digestive tract, the mouth, and the esophagus. There may be grayish-white, loosely attached, plaque-like areas on the internal surfaces of the crop in wild birds. Discoid, heightened ulcerative nodules that seem like rose clusters can also be present within the crop, displaying a textural appearance of a “Turkish bath towel” or “curds” due to the thickening of the crop’s cut surface. Pseudomembranes, areas of necrosis, and the accumulation of considerable tissue debris are the possible lesions observable in other areas of the upper digestive tract [16]. Fienners (1967) reported tongue lesions in gorillas (*Gorilla gorilla*) and woolly monkeys (*Lagothrix lagothricha*), whereas lesions occurred in the tongue, oral mucosa, intestine, liver, and lungs of a capuchin monkeys (*Cebus capucinus*) [57]. Buccal lesions in baboons (*Papio* sp.) kept at a zoological park in Paris yielded *Candida* [76]. A rough, yellow, and adherent pseudomembrane lining the entire esophagus was observed during a necropsy in a Rhesus monkey (*Macaca mulatta*) with thrush [36]. Post-mortem findings in a baby chimpanzee (*Pan troglodytes*) diagnosed with thrush included yellow and gelatinous pericardial fat at the base of the heart and a yellowish-white material adhered closely to the esophagus [58]. Wikse et al. (1970) and Patterson et al. (1974) isolated *Candida* from nine monkeys, including three spider monkeys (*Ateles* sp.), with enterocolitis [77,78]. The authors also reported a fungal invasion in the epithelium of the tongue, oral cavity, esophagus, colon, and nails. Furthermore, they grossly observed white patches or ulcers of the mucosa in the anterior alimentary tract whereas a thick pseudomembrane was seen on the colon, which was laden with *Candida*. The participation of the nails as in typical *Candida* onychomycosis has also been outlined [76].

The whole body of an 11-month-old Kodiak bear (*Ursus arctos middendorffi*) was covered with dense hyperkeratotic scales which appeared as round, partly confluent, and sharply demarcated skin lesions, with conical to 15 mm high skin tumors placed on the nasal cones. The horny layer of the paws was not far from being entirely detached and was smoothly removable. A basal hyperkeratosis was present [59]. McCullough et al. (1977) observed the involvement of nasal, pharyngeal, and intestinal mucosal surfaces as well as pharyngeal lymphadenopathy in a capuchin monkey (*Cebus capucinus*) with spontaneous candidosis [79]. A continuous nasal exudation and weight loss that led to fatality were also recorded. Saëz et al. (1978) noted buccal *Candida* infection and intestinal invagination in carcasses of captive baboons (*Papio* sp.) [61]. Glossitis was reported as the most common form of *Candida* infection in wild animals [76]. In a sloth (*Bradypus* sp.) with candidiasis, Berrocal (2017) observed gastritis with multiple ulcerative necrotic foci, and the anterior pyloric part had multiple growths similar to volcanic craters and extreme necrotic bronchopneumonia [80]. Additionally, the diaphragmatic lobe of the right lung was congested, and the left lung had thickened interlobular septa with the associated trachea and bronchus filled with frothy exudates.

#### 3.4.4. Histopathological Findings

A necrotic esophageal epithelium with many blastospores was noticed in the case of the baby chimpanzee (*Pan troglodytes*) diagnosed with thrush along with pseudohyphae that are also seen in all cases of *Candida*-associated enterocolitis in monkeys [58,76,77]. Upon the spontaneous candidosis in a capuchin monkey (*Cebus capucinus*), granulomatous inflammation with blastospores and pseudohyphae was reported in some organs [79]. Generalized chronic cutaneous candidiasis and extensive esophageal and gastric ulcerations were observed in two dolphins (*Delphinus delphis*) raised indoors [81]. Areas of hepatic focal necrosis and kidney stones were noted in a 2-year-old myna (*Acridotheres tristis*) with systemic candidiasis [75]. In addition, intestinal lesions (congestion, diffused hemorrhage, and goblet cell hyperplasia) were also observable. The organism appeared as masses of entangled pseudohyphae and budding yeast-like organisms in the liver and kidneys. Moreover, the fungus invaded the blood vessel wall and resulted in vasculitis characterized by an infiltration of the inflammatory cells (heterophils, macrophages, and lymphocytes).

Numerous microabscesses and granulomas (composed of eosinophils or Splendore–Hoeppli reaction material, pseudohyphae, and infrequently branching septate hyphae) were noticed in a senescent cheetah (*Acinonyx jubatus*) with candidiasis [63]. It was demonstrated that fluorescent antibody testing may reveal the branching septate hyphae of *Candida* spp. [80]. Stomach sections of the Linnaeus’s two-toed sloth (*Choloepus didactylus*) diagnosed with candidiasis revealed multiple necrotic centers resembling craters on the squamous epithelium of the prepyloric stomach, which even deeply reached the muscular layer of the mucosa [80]. There was a great deal of cellular debris on the necrotic tissue admixed with myriads of mycelial structures positive for Grocott and PAS stains [80].

#### 3.4.5. Hematological and Serum Biochemical Findings

The blood picture, in the case of a myna bird (*Acridotheres tristis*) with systemic candidiasis, revealed leukocytosis, heterophilia, and monocytosis while the serum showed increased activities of the liver enzymes, along with hyperproteinemia and hyperglobulinemia [75].

## 4. Histoplasmosis

Histoplasmosis, also called Darling’s or Spelunker’s disease, is a leading endemic mycosis around the world. Both immunocompromised and immunocompetent individuals can succumb to histoplasmosis [82,83,84]. Histoplasmosis is primarily a respiratory and systemic infection [82,85]. Natural infections of *Histoplasma* have been reported in captive and wild animals [84,86,87].

### 4.1. Etiology

Histoplasmosis is a zoonotic mycotic infection caused by *Histoplasma capsulatum*, a dimorphic fungus with two known varieties: *H. capsulatum* var. *capsulatum* and *H. capsulatum* var. *duboisii* [88,89,90]. *Histoplasma capsulatum* var. *duboisii* lives mostly in soil containing large amounts of bird or bat droppings [91]. *Histoplasma* has been isolated from several organs from bats and wild rodents [92,93]. Natural histoplasmosis was also detected in baboons (mostly sourced from West Africa) in France and USA [94]. Burek-Huntington et al. (2014) reported the presence of this fungus in sea otters (*Enhydra lutris*) in Alaska [95]. Whereas in the Amazon rainforest, histoplasmosis has been diagnosed in numerous animal species, including opossums (*Didelphis* spp.), armadillos (*Dasypus* spp.), rodents (*Proechimys* spp., *Agouti paca*), and sloths (*Choloepus didactylus*) [96].

### 4.2. Epidemiology

Histoplasmosis is a common disease in Africa, Asia, and Central and South America; however, it is a rare disease in Europe. There have been only few cases of histoplasmosis reported in Southern and Eastern Europe [97]. *Histoplasma* spp. thrives in high-nitrogen soil under humid environmental conditions [98]. *Histoplasma* infection is also very prevalent among wild (e.g., marsupials, rodents, armadillos, sloths, and bats) and domestic animals in endemic areas [3]. In Alaska, the main vectors of the disease are seabirds (black-legged kittiwakes—*Rissa tridactyla* and tufted puffins—*Fratercula cirrhata*) [99].

### 4.3. Diagnosis

A demonstration of the yeast-like *H. capsulatum* cells by direct microscopic examination using specific special staining techniques and an isolation of the fungi from clinical specimens (which is time-consuming) constitute the definitive methods for the diagnosis of histoplasmosis [100]. Alternative methods have been used as complementary diagnostic tools to detect anti-*Histoplasma* antibodies or *Histoplasma* antigens to thereby remove the time-consuming factor associated with a fungal culture [100,101]. Molecular methods with improved sensitivity are still under development [90,100]. ELISA tests (such as serum quantitative antigen tests) and radiology have been used with great success for diagnosing histoplasmosis [102]. Hemagglutination, the complement fixation test (CFT), a radioimmunoassay (RIA), etc., have also been developed as alternative serological techniques for detecting *H. capsulatum* var. *capsulatum* antibodies [47]. Other authors recommend nested PCR to highlight a 210 bp fragment specific for *H. capsulatum* [103].

### 4.4. Pathology

#### 4.4.1. Pathogenicity and Pathogenesis

There are speculations as to *Histoplasma’s* major portal of entry; however, it has been suggested that following inhalation, the fungus can be disseminated through the bloodstream to a favorable site such as the skin, bone, or lymph nodes [104,105]. Indeed, an erogenous transmission resulting in the deposition of fungal spores in the alveoli has been reported [106,107]. In addition, transcutaneous transmission via trauma and the possibility of transmission following insect bites have been suggested [106,107]. *H. capsulatum* yeasts, unlike other pathogens, can infect macrophages, survive antimicrobial activity, and proliferate as an intracellular pathogen. *H. capsulatum* yeasts receive protection from the extracellular proteins found in the lung surfactant by involving the β-integrin family of phagocytic receptors [108]. Moreover, *H. capsulatum* yeasts have been reported to conceal immunostimulatory β-glucans to avoid triggering the signaling receptors such as β-glucan receptor Dectin-1 [108]. *H. capsulatum* yeasts counteract phagocyte-produced reactive oxygen species (ROS) by expressing oxidative stress defense enzymes, including extracellular superoxide dismutase and catalase [108].

#### 4.4.2. Clinical Signs

Rhesus monkeys with histoplasmosis presented signs including coughing, anorexia, emaciation, and intermittent weakness followed by death [34]. An African pygmy hedgehog (*Atelerix albiventris*) with histoplasmosis showed signs of inappetence, weakness, lethargy, and weight loss [97]. In wild bats (*Mormoops megalophylla*), the disease often develops asymptomatically [109].

#### 4.4.3. Macroscopic Findings

The post-mortem lesions observed in a Rhesus monkey that died of histoplasmosis included miniature to large whitish nodules, cavitation, caseation, suppuration, and a blackish to greenish discoloration of the organs [34]. Emaciation, multiple nodulations, and ulcers were present in the skin. A badger (*Meles meles*) that died of histoplasmosis had pea to chestnut-sized flabby-to-firm lesions embedded in the skin [97]. Additionally, the drainage lymph nodes in the badger were moderately enlarged. Splenomegaly, hepatomegaly, generalized lymphadenopathy, and diffusely consolidated lungs were observed in the carcasses of two 6-month-old raccoons (*Procyon lotor*) that died of histoplasmosis [98].

#### 4.4.4. Histopathological Findings

Histoplasmosis infection is characterized by an infiltration of epithelioid cells, macrophages (with yeast-like bodies resembling empty red rings in their cytoplasm), multinucleated giant cells, and reticuloendothelial cells [110]. In the lung sections of Rhesus monkeys, multifocal to diffuse forms of calcified nodules with giant cells, epithelioid cells, and macrophages full of yeast-like spores were detected [34]. In several organs from raccoons (*Procyon lotor*), an extensive infiltration of macrophages laden with yeast cells was observed [98]. Diffused histiocytic inflammation with fibrosis and necrotic foci were also noted in the lungs, spleen, and intestinal sections of birds. In their liver and kidneys, marked bridging portal fibrosis and renal fibrosis were observed. In the lungs of these birds were thickened alveolar septa, filled alveolar spaces, random multifocal necrosis, variable alveolar septal fibrosis, and partial occlusion of larger pulmonary vessels. In the lymph nodes and the spleen, there was a prominently diffused infiltration by macrophages (laden with the fungi), thickened lymph node cortices, medullary and splenic cords, and mildly diffused interstitial fibrosis and necrotic foci. The enlarged splenic nodule had foci with hemorrhage and necrosis. In addition to a histiocytic infiltration and an invasion of organisms into the intestines, there were thickened lamina propria of the small intestine and colon mucosa with accompanying submucosal fibrosis. Moreover, noticeable thymic atrophy was also noticed in raccoons [98].

In sections of organs from a wild badger (*Meles meles*), Bauder et al. (2000) observed granulomas consisting of macrophages laden with yeast-like organisms, multinucleated giant cells, lymphocytes, plasma cells, and small clusters of granulocytes with connective tissue proliferation [97].

#### 4.4.5. Hematological and Serum Biochemical Findings

Clinicopathologic findings in African pygmy hedgehogs (*Atelerix albiventris*) with histoplasmosis included anemia, thrombocytopenia, leukopenia, hypoproteinemia, and hypoglycemia [87].

## 5. Cryptococcosis

Cryptococcosis has been reported in wildlife [111,112,113]. In fact, in Australia, cryptococcosis is a vital systemic fungal disease affecting mammals and amounting to more than 90% of fungal infection cases in mammals determined by culture and/or immunohistochemistry [114,115].

### 5.1. Etiology

*Cryptococcus neoformans*, the commonest suspect in cryptococcosis is an exogenic pathogen capable of causing lethal infections, especially in immunosuppressed individuals. *C. neoformans* has been isolated from gang cockatoos (*Cacatua* sp.), potoroos (*Potorous* sp.), sugar gliders (*Petaurus breviceps*), and ferrets (*Mustela furo*) [115,116]. *Cryptococcus* *gattii* has been isolated from cases of cryptococcosis in koalas (*Phascolarctos cinereus*), Australian king parrots (*Alisterus scapularis*), corellas (*Licmetis* sp.), Major Mitchell’s cockatoos (*Lophochroa leadbeateri*), Gilbert’s potoroos (*Potorous gilbertii*/*Hypsiprymnus gilbertii*), quokkas (*Setonix brachyurus*), gliders (*Petaurus* sp.), Eastern water skinks (*Eulamprus quoyii*), and ferrets (*Mustela furo*) [115].

### 5.2. Epidemiology

The inhalation of infective yeast cells results in a primary respiratory tract infection of the lungs followed by hematogenous dissemination, especially to the brain and spinal cord, skin, bones, joints, lymph nodes, and other internal organs [117,118]. Cryptococcosis has been reported in several wild animals, including Marmoset monkeys (*Callithrix jacchus*), Rhesus monkeys (*Macaca mulatta*), squirrel monkeys (*Saimiri sciureus*), shrews (*Sorex araneus*), marmosets (*Callithrix jacchus*), Eastern gray squirrels (*Sciurus carolinensis*), foxes (*Vulpes vulpes*), dolphins (*Delphinus delphis*), cheetahs (*Acinonyx jubatus*), Spinner dolphins (*Stenella longirostris*), koalas (*Phascolarctos cinereus*), ferrets (*Mustela furo*), California sea lions (*Zalophus californianus*), gorillas (*Gorilla gorilla*), Eastern water skinks (*Eulamprus quoyii*), and harbor porpoises (*Phocoena phocoena*) [22,111,112,118,119,120,121,122,123,124,125,126,127,128,129,130,131].

### 5.3. Diagnosis

The diagnosis of cryptococcosis is conducted by an isolation of the fungus; histochemistry using India ink, Nigrosin or Romanowski, and PAS stains; and immunohistochemistry [47,114,115]. Indirect fluorescent antibody test (FAT) and PCR can also be used in the diagnosis of cryptococcosis [47]. DNA fingerprinting and microsatellite analysis have also been used for diagnosing cryptococcosis [116].

### 5.4. Pathology

#### 5.4.1. Pathogenicity and Pathogenesis

Cryptococcosis presents in three forms: cutaneous, pulmonary, and meningococcal [132,133]. It causes cryptococcomas (cryptococcal granulomas) within parenchymatous organs [132,133]. The virulence attributes of *Cryptococcus* species include a polysaccharide capsule, melanin, mannitol production, the production of extracellular lipases, proteases, urease, superoxide dismutases, phospholipases, proteases, glutathione peroxidase, phosphatases, and DNases [47,90,134].

*Cryptococcus* is mainly an environmental organism, and it is acquired by the inhalation of spores. The infected host’s lungs (in which the organism becomes lodged in the alveoli) are the first point of contact of *C. neoformans* [47,135]. In animals, *Cryptococcus* tweaks the host’s adaptive immune response by inhibiting T-cell activation and the induction of T-cell apoptosis resulting in immunosuppression. In this state of depressed host immunity, the spores are revived and then spread into the different organs [47]. In affected animals, cryptococcosis is mostly restricted to the upper respiratory tract; however, the infection may extend locally to the central nervous system (CNS) (typically via the cribriform plate) or lower respiratory tract [136]. It may also spread through a hematogenous route. The hematogenous spread results in the multiplication inside the organism of the macrophages to produce a “cryptococcal phagosome”. This phagosome lyses the macrophages to release daughter yeast cells that spread to other organs [47,116,136].

There are differences in the pathogenesis of cryptococcosis in different animal species [116]. The reason for this variation is yet to be fully understood. Nevertheless, a variation in the size and complexity of nasal turbinate structures, the efficiency of the mucociliary clearance mechanisms and cough reflex, the length of the trachea, or behavioral exposure to different types of infectious propagules, may be responsible for the differences [116,137].

#### 5.4.2. Clinical Signs

The clinical manifestation of cryptococcosis in wild animals such as snakes (*Serpentes* sp.), koalas (*Phascolarctos cinereus*), ferrets (*Mustela furo*), and porpoises (*Phocoena phocoena*) has thus far been restricted to the lungs and central nervous system [138]. In ferrets, the eyes, gastrointestinal tract, and respiratory system have been affected [130,138]. Rhesus monkeys (*Macaca mulatta*) diagnosed with cryptococcosis had a history of mild pyrexia, dyspnea, and cough [119]. Roussilhon et al. (1987) associated the mortality of an old female squirrel monkey with cryptococcosis characterized by a tumor-like growth in the lungs [120]. Bradley et al. (1997) recorded the acute onset of dyspnea, depression, and lethargy in a juvenile female koala with cryptococcosis [139]. Tell et al. (1997) observed emaciation, shivering, dyspnea, and neurologic signs in shrews (*Soricidae*) from which *C. neoformans* was recovered [22]. A male Atlantic bottlenose dolphin (*Tursiops truncatus*) with a *Cryptococcus neoformans* var. *gattii* infection showed bronchopneumonia associated with pleuritis [123]. The dolphin had tachypnoea, transient dyspnea, mild tachycardia and multiple hyperechoic nodules, a consolidated lung, and thickened pleura that were observed post-mortem [123]. Polo Leal et al. (2010) and Illnait-Zaragozı et al. (2011) recorded weight loss, fatigue, asthenia, anorexia, and dyspnea with an excessive nasal secretion in cheetahs (*Acinonyx jubatus*) suffering cryptococcosis [124,140]. Morera et al. (2011) observed lymphadenopathy and acute bilateral blindness in a ferret with cryptococcosis, while Mischnik et al. (2014) reported that an old female gorilla (*Gorilla gorilla*) with disseminated cryptococcosis was lethargic, emaciated, and had a productive cough [128,131].

#### 5.4.3. Macroscopic Findings

Generalized lymphadenopathy and severe emaciation were observed in a koala with disseminated cryptococcosis [47]. Additionally, pale skeletal muscle, hydrothorax, pale lungs, wet and pale myocardium, and an abdominal cavity filled with clear and straw-colored fluid were observed in the koala. Furthermore, the liver edges of the koala were pale, friable, rubbery, and had whitish nodules of about 1mm in diameter that were randomly distributed throughout the splenic parenchyma [139]. In ferrets with cryptococcosis, rhinitis and the abscessation of the right retropharyngeal lymph node were observed [116]. In other ferrets with generalized cryptococcosis, the lower respiratory tract and intestine were primarily affected while pneumonia, pleurisy, and the involvement of the mediastinal lymph node, liver, and lung were common findings [116]. A harbor porpoise (*Phocoena phocoena*) was diagnosed with extensively scavenged, firm, and nodular lungs, and a sectioned surface exuding a clear or mucinous discharge [129]. The carcass of an Atlantic bottlenose dolphin (*Tursiops truncatus*) with cryptococcosis revealed bronchopneumonia with pleuritis, pulmonary lymphadenopathy, and diffused consolidated granulomatous lesions diffusely distributed in both lungs [123]. Millward and Williams (2005) noted a pulmonary granuloma and meningomyelitis in a free-ranging cheetah with cryptococcosis [141].

#### 5.4.4. Histopathological Findings

A histological section of the lungs of a male Rhesus monkey (*Macaca mulatta*) with cryptococcosis revealed widespread mononuclear cell infiltration along with cryptococcosis cells [119]. The presence of *Cryptococcus* and multiple foci of fungi in pulmonary and glandular localizations within the thoracic cavity were observed in sections of tissues from squirrel monkeys (*Saimiri* sp.) [120]. Juan-Salles et al. (1998) observed acute diffuse fibrin necrotizing enteritis, granulomatous endolymphangitis of the intestinal and mesenteric lymphatic vessels, multifocal granulomatous and necrotizing hepatitis, and mesangial nephropathy in sections of tissues from a marmoset (*Callithrix jacchus*) with cryptococcosis [122]. Bolton et al. (1999) observed pulmonary cryptococcosis and extensive meningoencephalomyelitis in sections of the lungs and brain from a cheetah with cryptococcosis [118]. In the case of a bottlenose dolphin (*Tursiops truncatus*), numerous spherical to ellipsoidal, mucicarmine-positive, encapsulated cells resembling *C. neoformans* were noticed in the lung sections [118].

Brain sections from an elk (*Cervus canadensis*) with cryptococcosis revealed multifocal to coalescing inflammatory nodules with necrotic areas surrounded by epithelioid macrophages, lymphocytes, plasma cells, and scattered multinucleated giant cells in the diencephalon, mesencephalon, and brain stem [142]. Many round extracellular yeasts with a thick capsule were observed. Lymphocytes and plasma cells infiltrated the adjacent neutrophil resulting in spongiosis. Occasionally, the Virchow Robin space was swollen, and perivascular cuffing was present. Moreover, the leptomeninges were thickened by cellular (lymphocytes, plasma cells, and macrophages) infiltration.

In sections of a lymph node biopsy from ferrets (*Mustela furo*), Morera et al. (2011) observed pyogranulomatous lymphadenitis with an intralesional yeast consistent with *Cryptococcus* species [128]. Microscopically, the lungs of a harbor porpoise (*Phocoena phocoena*) had granulomatous and pyogranulomatous infiltrates with many yeasts while its mediastinal lymph nodes showed mild granulomatous inflammation. The lymph nodes were encapsulated and partially replaced with intracellular and extracellular multilobulated yeast aggregates that were also encapsulated. Around the periphery of these yeast aggregates, the presence of macrophages, lymphocytes, and fewer neutrophils was noted. Yeasts cells were found in the amniotic fluid and interspersed within the chorioallantoic villi and the submucosal vasculature of the placenta. Mild multifocal nonsuppurative myocarditis was also detected [129].

## 6. Penicilliosis

Penicilliosis is an emerging infectious disease produced by *Talaromyces* (*Penicillium*) *marneffei*, which is a major pathogenic and thermally dimorphic fungus causing systemic mycosis [143,144,145].

### 6.1. Etiology

*Penicillium marneffei*, the etiologic agent of this mycotic disease, is a member of the family *Trichocomaceae* [144].

### 6.2. Epidemiology

*P. marneffei* infection is endemic in tropical regions, especially in Southeast Asia [144]. The predictive factors controlling the seasonal incidence of *P. marneffei* infection are unknown [146], although a high prevalence of infection has been recorded among bamboo rats of the genera *Rhizomys* and *Cannomys* (the only known animals where natural infection occurs) suggesting that these wild rodents are a key part of the *P. marneffei* life cycle [147]. The fungus was first isolated from the hepatic lesions of a bamboo rat (*Rhizomys sinensis*) that had died from the infection in 1956 [148]. Subsequently, it was documented that bamboo rats and the soil from their burrows were important enzootic and environmental (natural) reservoirs of *P. marneffei* [148,149,150]. However, penicilliosis is still rare in animals [151].

### 6.3. Diagnosis

A laboratory diagnosis of *P. marneffei* infection requires the microscopic affirmation of *P. marneffei* yeast in the infected tissue and the culture of the fungus from clinical specimens. *P. marneffei* is a unicellular organism and microscopically appears as round to oval cells, unlike other species in this genus. *P. marneffei* has a thermal dimorphism. This ability to grow as a mycelium at 25 °C and as yeast at 37 °C is the organism’s principal virulence factor [144]. The use of histopathology in the diagnosis of *P. marneffei* requires the use of special histochemical stains such as Grocott methenamine silver and PAS stains for a diagnosis. ELISA and PCR can also be used to detect these fungi in clinical specimens of wild animals [47].

### 6.4. Pathology

#### 6.4.1. Pathogenicity and Pathogenesis

An important risk factor in the spread of penicilliosis is soil exposure, especially during the rainy season. Genotyping studies have revealed that the transmission of *P. marneffei* may occur from rodents to humans or that rodents and humans are co-infected by common environmental sources. Further gene molecular studies found several genes involved in fungal germination, conidiogenesis, hyphal development, and yeast cell polarity. Several functionally important genes, such as the malate synthase and catalase-peroxidase protein-encoding genes, have been identified as being upregulated in the yeast phase and these play important role in the pathogenesis of this fungus, although more studies will be required to confirm these findings [144]. Upon entry into the body of the host, *P. marneffei* is transformed into yeast cells and spreads around the body in a hematogenous manner. They survive within phagocytes such as macrophages from which they spread all over the body, followed by fatal necrotizing reactions and histiocyte infiltration [47].

#### 6.4.2. Macroscopic Findings

Penicilliosis in a female captive-born Congo African grey parrot (*Psittacus erithacus*) showed the following gross lesions: emaciation; a dense yellowish fluid in the nares, mouth cavity, esophagus, and crop; and a moderate amount of ascites in the coelom. The intestine was filled with gray-whitish to yellowish fluid. There were multifocal yellowish-white nodules in the liver and left kidney. A yellowish exudate extravasated after sectioning the nodules. The lungs showed pulmonary edema with focal areas of large yellow-greenish nodules on the dorsum of the lungs. The section of the lung parenchyma presented grossly normal areas scattered with yellowish septa and yellow material inside the bronchial and tracheal lumen. The foci were alike in the thoracic cranial air sacs [151].

#### 6.4.3. Histopathological Findings

The histopathology of a captive-born Congo African grey parrot with penicilliosis revealed mature multifocal granulomas, typically caseous, with necrotic centers where several septate hyphae, as well as macrophages and giant cells, were detected in lung, liver, and kidney samples. The Grocott staining revealed black septate fungal hyphae with conidia [151].

## 7. Conclusions

Zoonotic fungal diseases create social and occupational hazards for humans that are in close contact with infected animals. However, although the universal burden of zoonotic fungal diseases is on a continuous increase, the public health attention accorded to them in developing countries is paltry. Besides dermatophytoses, which are prevalent among human and animal populations, many these zoonotic fungal diseases are overlooked and may thus be misdiagnosed because of physicians’ and veterinarians’ lack of experience and understanding [89,152]. Thus, there is a necessity to promote an increased degree of awareness of zoonotic fungal diseases around the world and especially those originating from wildlife. Therefore, they should be given preferential attention by relevant animal and human health authorities through smart but vigorous public literacy programs, and the One-Health approach should be globally endorsed to conquer the health threats that these zoonotic mycoses pose [89]. Considering the potential for zoonotic transmission of these mycoses to humans, especially with regard to occupational transmission among pet owners, veterinarians, herdsmen, agricultural workers, and zookeepers, there is a compelling need for continuous research into zoonotic mycoses [89]. In conclusion, mycoses in wildlife are of veterinary importance and possess zoonotic potential. Thus, this review highlights the etiology, epidemiology, diagnosis, and pathology of some important mycoses of wild animals to create the impetus to understand them and to further focus the attention of veterinarians, physicians, public health specialists, zookeepers, and wild pet owners toward these re-emerging and emerging mycoses in wildlife, which similar to their zoonotic bacterial and viral counterparts, could cause devastating effects in both humans and domestic/wild animals.

## Data Availability

Not applicable.
